# Supplementary material for: Pyrethroid susceptibility reversal in Aedes aegypti: A longitudinal study in Tapachula, Mexico
Source: PLoS Negl Trop Dis. 2024 Jan 2;18(1):e0011369. doi: 10.1371/journal.pntd.0011369 (PMC10786364; doi:10.1371/journal.pntd.0011369)
Supplement: S1 Table — Larvae were collected from each cemetery on 2016’s wet season. The municipality name and geographical coordinates are provided. (DOCX) [file pntd.0011369.s001.docx]

**S1_Table. Location of *Aedes aegypti* cemetery collections in the coast of Chiapas.** Larvae were collected from each cemetery on September 2016. The municipality name and geographical coordinates are also provided in Fig 2.

| **Cemetery (panteon)** | **Municipality** | **Latitude** | **Longitude** |
| --- | --- | --- | --- |
| S1 | Huixtla | 15.136806 | 92.452194 |
| S2 | Mapastepec | 15.445222 | 92.901222 |
| S3 | Mazatan | 14.867544 | 92.449967 |
| S4 | Motozintla | 15.365367 | 92.252292 |
| S5 | Pijijiapan | 15.684753 | 93.203253 |
| S6 | Puerto Madero | 14.722694 | 92.427417 |
| S7 | Ciudad Hidalgo | 14.685778 | 92.148889 |
| S8 | Tapachula (Jardin) | 14.894917 | 92.249056 |
| S9 | Tapachula (Municipal) | 14.90425 | 92.270306 |
