# Supplementary material for: Pyrethroid susceptibility reversal in Aedes aegypti: A longitudinal study in Tapachula, Mexico
Source: PLoS Negl Trop Dis. 2024 Jan 2;18(1):e0011369. doi: 10.1371/journal.pntd.0011369 (PMC10786364; doi:10.1371/journal.pntd.0011369)
Supplement: S2 Table — RR’s were obtained by bottle bioassays. Analyses were ran separately for each pyrethroid (permethrin and deltamethrin) and each collection time point, including 2018, 2019, and 2020. S = September and M = March. p values above 0.05 are considered non-significant. N = the number of pairwise comparisons between sites in the specific distance class. (DOCX) [file pntd.0011369.s002.docx]

**Supplementary Table S2. Moran's I statistic showing autocorrelation coefficients between the pyrethroid RR's and the geographic distance between 24 *Aedes aegypti* collection sites in Tapachula, Mexico.** RR’s were obtained by bottle bioassays. Analyses were ran separately for each pyrethroid (permethrin and deltamethrin) and each collection time point, including 2018, 2019, and 2020. S = September and M= March. *p* values above 0.05 are considered non-significant. N= the number of pairwise comparisons between sites in the specific distance class.

|  | **Permethrin** | | | |  | **Deltamethrin** | | | | |
| --- | --- | --- | --- | --- | --- | --- | --- | --- | --- | --- |
|  | Distance class | Autocorrelation coefficient | *p* value | n |  |  | Distance class | Autocorrelation coefficient | *p* value | n |
| 2018-S (62-months) |  |  |  |  |  |  |  |  |  |  |
| [1,] | 740.53 | -0.019 | 0.429 | 64 |  | [1,] | 740.53 | 0.215 | 0.065 | 64 |
| [2,] | 1769.76 | -0.081 | 0.594 | 82 |  | [2,] | 1769.76 | 0.018 | 0.341 | 82 |
| [3,] | 2798.99 | 0.122 | 0.114 | 104 |  | [3,] | 2798.99 | -0.038 | 0.485 | 104 |
| [4,] | 3828.22 | -0.226 | 0.909 | 94 |  | [4,] | 3828.22 | 0.100 | 0.151 | 94 |
| [5,] | 4857.45 | -0.070 | 0.562 | 68 |  | [5,] | 4857.45 | -0.203 | 0.823 | 68 |
| [6,] | 5886.69 | -0.317 | 0.961 | 78 |  | [6,] | 5886.69 | -0.321 | 0.960 | 78 |
| [7,] | 6915.92 | 0.437 | **0.020*** | 32 |  | [7,] | 6915.92 | -0.515 | 0.963 | 32 |
| [8,] | 7945.15 | 0.295 | 0.103 | 10 |  | [8,] | 7945.15 | -0.089 | 0.424 | 10 |
| [9,] | 8974.38 | -0.016 | 0.142 | 10 |  | [9,] | 8974.38 | -0.008 | 0.275 | 10 |
| [10,] | 10003.61 | -0.047 | 0.154 | 10 |  | [10,] | 10003.61 | -0.001 | 0.230 | 10 |
|  |  |  |  |  |  |  |  |  |  |  |
| 2019-M (68-months) |  |  |  |  |  |  |  |  |  |  |
| [1,] | 740.53 | -0.054 | 0.510 | 64 |  | [1,] | 740.53 | 0.136 | 0.143 | 64 |
| [2,] | 1769.76 | -0.146 | 0.743 | 82 |  | [2,] | 1769.76 | 0.038 | 0.294 | 82 |
| [3,] | 2798.99 | -0.129 | 0.730 | 104 |  | [3,] | 2798.99 | -0.251 | 0.931 | 104 |
| [4,] | 3828.22 | -0.085 | 0.618 | 94 |  | [4,] | 3828.22 | -0.197 | 0.862 | 94 |
| [5,] | 4857.45 | -0.324 | 0.949 | 68 |  | [5,] | 4857.45 | -0.371 | 0.971 | 68 |
| [6,] | 5886.69 | 0.151 | 0.105 | 78 |  | [6,] | 5886.69 | 0.002 | 0.381 | 78 |
| [7,] | 6915.92 | 0.012 | 0.361 | 32 |  | [7,] | 6915.92 | -0.147 | 0.609 | 32 |
| [8,] | 7945.15 | -0.162 | 0.496 | 10 |  | [8,] | 7945.15 | 0.453 | 0.078 | 10 |
| [9,] | 8974.38 | -0.043 | 0.152 | 10 |  | [9,] | 8974.38 | -0.010 | 0.151 | 10 |
| [10,] | 10003.61 | -0.506 | 0.969 | 10 |  | [10,] | 10003.61 | -0.420 | 0.942 | 10 |
|  |  |  |  |  |  |  |  |  |  |  |
| 2019-S (74-months) |  |  |  |  |  |  |  |  |  |  |
| [1,] | 797.71 | 0.079 | 0.232 | 68 |  | [1,] | 797.71 | 0.169 | 0.109 | 68 |
| [2,] | 1941.30 | 0.134 | 0.116 | 80 |  | [2,] | 1941.30 | 0.041 | 0.282 | 80 |
| [3,] | 3084.89 | -0.221 | 0.915 | 104 |  | [3,] | 3084.89 | -0.096 | 0.652 | 104 |
| [4,] | 4228.48 | 0.006 | 0.371 | 78 |  | [4,] | 4228.48 | 0.008 | 0.368 | 78 |
| [5,] | 5372.07 | -0.322 | 0.965 | 80 |  | [5,] | 5372.07 | -0.029 | 0.445 | 80 |
| [6,] | 6515.66 | 0.131 | 0.150 | 64 |  | [6,] | 6515.66 | -0.093 | 0.608 | 64 |
| [7,] | 7659.25 | -0.685 | 0.902 | 12 |  | [7,] | 7659.25 | -0.099 | 0.424 | 12 |
| [8,] | 8802.84 | -0.206 | 0.532 | 10 |  | [8,] | 8802.84 | -0.042 | 0.301 | 10 |
| [9,] | 9946.43 | -0.029 | 0.252 | 10 |  | [9,] | 9946.43 | -0.091 | 0.311 | 10 |
|  |  |  |  |  |  |  |  |  |  |  |
| 2020-M (80-months) |  |  |  |  |  |  |  |  |  |  |
| [1,] | 740.53 | 0.052 | 0.277 | 64 |  | [1,] | 740.53 | 0.024 | 0.330 | 64 |
| [2,] | 1769.76 | 0.150 | 0.103 | 82 |  | [2,] | 1769.76 | -0.436 | 0.995 | 82 |
| [3,] | 2798.99 | 0.026 | 0.309 | 104 |  | [3,] | 2798.99 | 0.127 | 0.106 | 104 |
| [4,] | 3828.22 | -0.071 | 0.578 | 94 |  | [4,] | 3828.22 | 0.089 | 0.167 | 94 |
| [5,] | 4857.45 | -0.192 | 0.808 | 68 |  | [5,] | 4857.45 | -0.241 | 0.879 | 68 |
| [6,] | 5886.69 | -0.066 | 0.552 | 78 |  | [6,] | 5886.69 | 0.046 | 0.277 | 78 |
| [7,] | 6915.92 | -0.602 | 0.980 | 32 |  | [7,] | 6915.92 | 0.140 | 0.185 | 32 |
| [8,] | 7945.15 | -0.270 | 0.601 | 10 |  | [8,] | 7945.15 | -0.059 | 0.394 | 10 |
| [9,] | 8974.38 | -0.020 | 0.117 | 10 |  | [9,] | 8974.38 | -0.007 | 0.233 | 10 |
| [10,] | 10003.61 | 0.000 | 0.059 | 10 |  | [10,] | 10003.61 | -0.182 | 0.457 | 10 |
|  |  |  |  |  |  |  |  |  |  |  |
| 2020-S (86-months) |  |  |  |  |  |  |  |  |  |  |
| [1,] | 537.36 | 0.078 | 0.247 | 54 |  | [1,] | 525.91 | 0.247 | 0.059 | 52 |
| [2,] | 1587.98 | 0.037 | 0.289 | 84 |  | [2,] | 1577.73 | -0.135 | 0.743 | 96 |
| [3,] | 2638.60 | -0.270 | 0.933 | 94 |  | [3,] | 2629.56 | -0.312 | 0.973 | 102 |
| [4,] | 3689.21 | -0.217 | 0.905 | 100 |  | [4,] | 3681.38 | -0.001 | 0.376 | 106 |
| [5,] | 4739.83 | 0.143 | 0.123 | 72 |  | [5,] | 4733.20 | 0.233 | 0.038 | 78 |
| [6,] | 5790.45 | -0.078 | 0.581 | 78 |  | [6,] | 5785.02 | -0.047 | 0.510 | 82 |
| [7,] | 6841.07 | 0.233 | 0.079 | 42 |  | [7,] | 6836.85 | -0.081 | 0.552 | 52 |
| [8,] | 7891.68 | 0.048 | 0.251 | 10 |  | [8,] | 7888.67 | -0.518 | 0.825 | 10 |
| [9,] | 8942.30 | -0.525 | 0.931 | 8 |  | [9,] | 8940.49 | -0.087 | 0.343 | 10 |
| [10,] | 9992.92 | -0.016 | 0.254 | 10 |  | [10,] | 9992.32 | -0.119 | 0.427 | 12 |
