# Supplementary material for: Pyrethroid susceptibility reversal in Aedes aegypti: A longitudinal study in Tapachula, Mexico
Source: PLoS Negl Trop Dis. 2024 Jan 2;18(1):e0011369. doi: 10.1371/journal.pntd.0011369 (PMC10786364; doi:10.1371/journal.pntd.0011369)
Supplement: S3 Table — Independent variables include the insecticide concentration (log(concentration)), site (24 sites), time in months (after PYR discontinuation). Estimates, standard error, z-value, probability and change are shown for each variable. (DOCX) [file pntd.0011369.s003.docx]

**S3_Table.** **Summary of generalized linear model estimates for A) Permethrin and, B) Deltamethrin.** Independent variables include the insecticide concentration (log), site (24 sites), time in months (after PYR discontinuation). Estimates, standard error, z-value, probability, and change are shown for each variable. Significance *** = p *value* <0.001, ** = 0.001 < p *value* < 0.01; and * = 0.01 < p *value* < 0.05

A) Permetrhin

| Variable | Estimate | Std. Error | z value | Pr(>\|z\|) | Significance | Change |
| --- | --- | --- | --- | --- | --- | --- |
| Intercept | -7.348 | 0.119 | -61.779 | < 2e-16 | *** | 0.001 |
| Log(concentration) | 2.098 | 0.019 | 109.040 | < 2e-16 | *** | 8.148 |
| Month | 0.015 | 0.001 | 14.167 | < 2e-16 | *** | 1.015 |
| Site_NE2 | -1.196 | 0.100 | -11.935 | < 2e-16 | *** | 0.302 |
| Site_NE3 | -0.074 | 0.104 | -0.715 | 0.474877 |  | 0.929 |
| Site_NE4 | -0.772 | 0.105 | -7.351 | 1.97E-13 | *** | 0.462 |
| Site_NE5 | -0.582 | 0.100 | -5.803 | 6.51E-09 | *** | 0.559 |
| Site_NE6 | -0.966 | 0.097 | -9.977 | < 2e-16 | *** | 0.381 |
| Site_NW1 | -0.826 | 0.096 | -8.612 | < 2e-16 | *** | 0.438 |
| Site_NW2 | -1.372 | 0.084 | -16.345 | < 2e-16 | *** | 0.254 |
| Site_NW3 | -0.430 | 0.093 | -4.629 | 3.68E-06 | *** | 0.651 |
| Site_NW4 | -0.824 | 0.098 | -8.437 | < 2e-16 | *** | 0.438 |
| Site_NW5 | 0.192 | 0.090 | 2.127 | 0.033394 | * | 1.212 |
| Site_NW6 | -0.599 | 0.096 | -6.266 | 3.71E-10 | *** | 0.550 |
| Site_SE1 | -0.659 | 0.100 | -6.613 | 3.77E-11 | *** | 0.517 |
| Site_SE2 | -0.072 | 0.100 | -0.716 | 0.473898 |  | 0.931 |
| Site_SE3 | -1.125 | 0.085 | -13.221 | < 2e-16 | *** | 0.325 |
| Site_SE4 | -0.664 | 0.102 | -6.516 | 7.24E-11 | *** | 0.515 |
| Site_SE5 | 0.212 | 0.104 | 2.041 | 0.041232 | * | 1.236 |
| Site_SE6 | -0.203 | 0.098 | -2.067 | 0.038763 | * | 0.816 |
| Site_SW1 | -0.115 | 0.083 | -1.375 | 0.169204 |  | 0.892 |
| Site_SW2 | -0.047 | 0.093 | -0.504 | 0.613916 |  | 0.954 |
| Site_SW3 | -0.404 | 0.097 | -4.172 | 3.02E-05 | *** | 0.668 |
| Site_SW4 | -0.321 | 0.098 | -3.284 | 0.001024 | ** | 0.726 |
| Site_SW5 | -1.255 | 0.103 | -12.138 | < 2e-16 | *** | 0.285 |
| Site_SW6 | -0.284 | 0.085 | -3.351 | 0.000805 | *** | 0.753 |

B) Deltamethrin

| Variable | Estimate | Std. Error | z value | Pr(>\|z\|) | Significance | Change |
| --- | --- | --- | --- | --- | --- | --- |
| Intercept | -6.782 | 0.107 | -63.178 | < 2e-16 | *** | 0.001 |
| Log(concentration) | 1.498 | 0.014 | 110.203 | < 2e-16 | *** | 4.474 |
| Month | 0.081 | 0.001 | 65.266 | < 2e-16 | *** | 1.084 |
| Site_NE2 | -0.951 | 0.087 | -10.939 | < 2e-16 | *** | 0.387 |
| Site_NE3 | -0.272 | 0.091 | -2.993 | 0.002761 | ** | 0.762 |
| Site_NE4 | -0.594 | 0.098 | -6.073 | 1.25E-09 | *** | 0.552 |
| Site_NE5 | -0.836 | 0.091 | -9.203 | < 2e-16 | *** | 0.434 |
| Site_NE6 | -0.944 | 0.088 | -10.762 | < 2e-16 | *** | 0.389 |
| Site_NW1 | -0.535 | 0.086 | -6.211 | 5.27E-10 | *** | 0.586 |
| Site_NW2 | -0.312 | 0.077 | -4.04 | 5.34E-05 | *** | 0.732 |
| Site_NW3 | 0.072 | 0.084 | 0.853 | 0.39366 |  | 1.075 |
| Site_NW4 | 0.000 | 0.088 | 0.002 | 0.998245 |  | 1.000 |
| Site_NW5 | 0.424 | 0.083 | 5.132 | 2.87E-07 | *** | 1.528 |
| Site_NW6 | 0.173 | 0.084 | 2.067 | 0.03875 | * | 1.189 |
| Site_SE1 | -0.930 | 0.090 | -10.284 | < 2e-16 | *** | 0.394 |
| Site_SE2 | 0.349 | 0.089 | 3.933 | 8.38E-05 | *** | 1.417 |
| Site_SE3 | -0.799 | 0.077 | -10.321 | < 2e-16 | *** | 0.450 |
| Site_SE4 | -0.309 | 0.090 | -3.454 | 0.000553 | *** | 0.734 |
| Site_SE5 | 0.292 | 0.090 | 3.25 | 0.001154 | ** | 1.338 |
| Site_SE6 | -0.185 | 0.093 | -1.996 | 0.045898 | * | 0.831 |
| Site_SW1 | -0.489 | 0.077 | -6.332 | 2.42E-10 | *** | 0.613 |
| Site_SW2 | 0.606 | 0.082 | 7.36 | 1.83E-13 | *** | 1.833 |
| Site_SW3 | -0.706 | 0.085 | -8.334 | < 2e-16 | *** | 0.493 |
| Site_SW4 | 0.392 | 0.086 | 4.571 | 4.85E-06 | *** | 1.481 |
| Site_SW5 | 0.014 | 0.093 | 0.153 | 0.878695 |  | 1.014 |
| Site_SW6 | -0.114 | 0.077 | -1.485 | 0.137452 |  | 0.892 |
