# Supplementary material for: Pyrethroid susceptibility reversal in Aedes aegypti: A longitudinal study in Tapachula, Mexico
Source: PLoS Negl Trop Dis. 2024 Jan 2;18(1):e0011369. doi: 10.1371/journal.pntd.0011369 (PMC10786364; doi:10.1371/journal.pntd.0011369)
Supplement: Attachment — Submitted filename: Response_to_reviewers.docx [file pntd.0011369.s005.docx]

**Dear editor,**

**Please see my response to the reviewer’s comments in blue.**

METHODS

**Reviewer #1:** -Number of replicates for each assay was not stated.

We agree, we have added the following sentence in line 196: “Between five and six insecticide concentrations were tested in triplicate”. We also added the range of insecticide concentrations used for the bottle bioassays to test field-collected colonies and the New Orleans susceptible strain.

-State the total number of mosquitoes used in bioassay and kdr genotyping

We agree, we have added this information in line 202: ‘Between 350 and 500 mosquitoes from each collection site were used to calculate the LC50 for permethrin and deltamethrin’.

For the genotyped mosquitoes we added in line 221: “Between 950 and 1250 individual mosquitoes were genotyped at each collection time. In total, 5,948 individual mosquitoes were genotyped at both loci during the study period (2016 to 2020).”

-F1/F2 mosquitoes were used. How did you give a blood meal to the mosquitoes?

We agree, we added the following information in line 169: ‘The females were blood feed on a rabbit following the guidelines of the Centro Nacional de Programas Preventivos y Control de Enfermedades (CENAPRECE) approved by the Ethical Commission of the Instituto Nacional de Salud Pública (CENAPRECE guidelines)’

**Reviewer #2:** This is in-depth longitudinal study on the Pyrethroid resistant level in Aedes aegypti using an IRM Strategy. It's hypotheses are clearly stated, the sample size adequate and methods are appropriate for the study.

Thank you

**Reviewer #3:** There are items that are used in analysis that are not described in the methods, particularly collection sites.   There is also confusion on what some terms are referring to.  In particular concentration of insecticide.  Does it refer to assay or field application on page 9.
When running assays that calculate RR the comparator (Rock strain in this study) should ALWAYS be run in real time with test populations.  Not periodically.

- We agree, we have made our best effort to clarify these observations throughout the manuscript.
- All the data presented in this study correspond to laboratory bioassays.
- We established mosquito colonies from larva collected in each of the 24 sites. The collections were made at different time points from 2016 to 2020.
- We agree, we used our reference susceptible strain, in this case New Orleans every time we ran a bioassay to test a collection site. We evaluated between 4 and 5 mosquito colonies for a particular insecticide in a day. A New Orleans bioassay was ran every testing day to determine resistance ratios. Overall, New Orleans had LC50’s ranging between 0.6 and 0.9 ug/bottle across the five years of study. This variation was accounted during the resistance ratio calculation.

RESULTS

Figures

**Reviewer #1:** Appropriate

**Reviewer #2:** Graphs could benefit with some indication of significant results but otherwise the results are clearly presented and sufficient.

We agree, we included the following information in the Fig 3 legend “Bars represent the 95% confidence intervals. Non-overlapping bars indicate the RR’s were significantly different. None of our colonies were equal to the New Orleans reference strain RR.”

**Reviewer #3:** There is a duplicate figure.  Figures 5 and Figure S1 are the same.  The only difference is 5 is separated into 2 panels and S1 combines them in 1 graph.  Other comments are in uploaded document.

We agree, we will remove Figure S1 from the manuscript.

Looking for geographic trends was hinted at but not clearly described.

We agree, now we include an autocorrelation analysis that yield non-significant results on line 328 ‘A Moran’s autocorrelation analysis was conducted using the methodology described in Solis Santoyo et al (2020) [32]. For each collection period, we tested whether pyrethroid resistance ratios (RR) were explained by geographic distance between sites. None of the analyses yielded significant correlation (data not shown). Accordingly to our previous study in Tapachula [32], lack of correlation imply that closer geographical collection sites do no share similar levels of pyrethroid resistance, and that resistance is rather explained by focal conditions at each site.’

Chi squares for LC50 calculations need to be added.
We agree, however it’s difficult to add ~280 rows of LC50 models in the manuscript. We have addressed this in line 210: “ Then, a Pearson goodness of fit test tested the adjustment of our data to the binary logistic regression model. The null hypothesis (Ho) assumed the observed mortality curve adjusts to a binary logistic regression model. Thus, we expected p values greater than 0.05 to accept the Ho. When the Ho was rejected, the bioassay was excluded and repeated. Our study includes data from bioassays that adjusted to the binary logistic regression model (p values > 0.05).‘

CONCLUSIONS

**Reviewer #1:** Detoxification mechanism was not studied and this limitation must be acknowledged.

We agree. We included in line 462 “**A limitation of our study is that we did not evaluate the role of detoxification mechanisms. However…”**

Editorial and data presentations

**Reviewer #1:** n/a
**Reviewer #2:** (No Response)
**Reviewer #3:** see attachment

Observations have been addressed. We appreciate the time to make observations.

**Summary and General Comments**

**Reviewer #1:** This is an interesting study and the findings could be useful in developing effective control measures against dengue. Prior to acceptance, please illustrate the method in details so that it can be replicated by the other researchers. Detoxification is the main resistance mechanism in Aedes but this was not investigated. Please acknowledge this limitation.

We agree, we added an ‘experimental design’ section in line 135. Also we have added details in our methods. Corrections and limitations have been added throughout the manuscript.

**Reviewer #2:** Overall, A well written and logical paper, highly valuable for the assessment of applied IRM strategies.
Thank you.

**Reviewer #3: see attachment**

153: Describe panteon data. Clarify preliminary sample sites and add to map.

We agree. This was added in line 160-165. Positions in the city were included on the map.

164: Were sites randomly selected based on 2016?

We agree, this is mention in line 175.

155: Whats 3 x 3 x3

We agree, we now mention ‘3 blocks x 3 blocks x 3 blocks’ in line 179.

179: If you are not going to address these here then delete. Save for paper which will include them.

We agree, insecticides not included in this study were removed

165: Is there a protocol number for the feeding?

We have included the following paragraph in line 169: ‘The females were blood feed on a rabbit following the guidelines of the Centro Nacional de Programas Preventivos y Control de Enfermedades (CENAPRECE) approved by the Ethical Commission of the Instituto Nacional de Salud Pública (CENAPRECE guidelines),’

184: X2 also needed to ensure data fits your model. If less 10% variation should repeat experiment.

We agree, we only included bioassay experiments that significantly adjusted to the binomial logistic regression (line 214). Description of the goodness of fit test was added on line 211.

188: strain should be used with every experiment. Was this to confirm stock still active?

Yes, we clarified that we conducted a LC50 calculation for New Orleans for each set of daily experiments in line 207.

Describe panteon collections and how used since you include data in Fig 3, also include in narrative.

We agree, we have added this information in line 160.

211: Concentration in assays or in field applications. Please clarify what you used.

We agree, we have clarified in the experimental design section (line 135) and throughout the manuscript.

**RESULTS**

231: Whole study time?

We agree. We have added the following graph ‘The goal of our study is to evaluate the changes in pyrethroid resistance in mosquito populations as a response to pyrethroid discontinuation from vector control programs. Our study measured the changes in resistance by comparing the LC50 between 2016 and 2020 across Tapachula, however, removal of pyrethroid occurred in 2013. In this section, we present the use of insecticides by vector control programs in the City of Tapachula from 2012 to 2020 with the goal of establishing the insecticide pressure over mosquito populations before and during our study.’

279: Chiapas: describe in methods and include as dots on map.

This has been addressed

293: Where these trends for sites based on quadrants what was selection to ….. and spraying since it seems by concentration you mean ain’t in assay.

We agree, we have added in line 294 ‘In our initial screening in 2016, permethrin RRs among collection sites ranged from 10- to 81-fold’

294: Without knowing what you define as concentration as would it surprise you that increasing a dose by 1 log would increase mortality.

We agree, we have clarified that concentration is in ug/bottle in the methods section. Also, we added the following information on line 328 “…..one-unit increase in one log of permethrin concentration (natural logarithm of concentration in µg) corresponded to an increase in the odds of dying of 814% (p value = 2E-16)”

254: Did you run NO at same time each time? Since using indirect measure of IR differences in rearing could have skewed the results. You have to run your comparator at same time

We agree, we have addressed this point in the methods section.

312: Quadrant or 2016 10 sites vs cemeteries?

We agree, we added in line 351 “In 2016, we screened ten sites in the city of Tapachula and seven sites along the coast of Chiapas (cemeteries).”

357: Most of diagnostic dose time should change not time itself if CDC assay done.

We agree, we corrected the sentence by “The results did not detect a decline in mortality with the CDC bottle bioassay”

361: They also measure two different things. It’s like comparing genotype to weight in one. Study and genotype to distance in the next

We agree, it’s our intention to compare the different method approaches.

365: Be clear here they did not use CDC time mortality assay but modified to be dose mortality assay. Don’t call it the cdc bottle bioassay but modify and CDC assay.

We agree, we have corrected this information.

Fig 3: add to methods

We agree, we have included in narrative.

513: Duplicate Figure with data instead of two

We agree, we have removed Fig S1.

Fig 2: Add panteon sites in map

This has been addressed.

Fig 3: Describe in narrative for methods, materials and results

Thanks. This has been added in line 292.

Fig 4: Don’t use an incrementing bar. Use discrete colors for each month. Please add to x axis concentration to each column

We agree, Fig 4 has been modified according to reviewer observations.

Fig 5:

Duplicate Fig S1 with i1016 and C1534. Separate vs combine delete one of these

We agree, we have removed Figure S1.

Supplementary Table S1: add to the map figure.

This has been added to Fig 2

Supplementary Table S2: Define *, **, ***

This has been corrected in Table legend.
